# Investigating electrochemical corrosion at Mg alloy-steel joint interface using scanning electrochemical cell impedance microscopy (SECCIM)

**DOI:** 10.1038/s41598-023-39961-2

**Published:** 2023-08-15

**Authors:** Venkateshkumar Prabhakaran, Lyndi Strange, Rajib Kalsar, Olga A. Marina, Piyush Upadhyay, Vineet V. Joshi

**Affiliations:** 1 Physical and Computational Sciences Directorate, Pacific Northwest National Laboratory, Richland, WA USA; 2 Energy and Environment Directorate, Pacific Northwest National Laboratory, Richland, WA USA

**Keywords:** Structural materials, Materials science, Nanoscale materials, Chemical engineering, Corrosion

## Abstract

Developing strategies to prevent corrosion at the interface of dissimilar metal alloys is challenging because of the presence of heterogenous distribution of galvanic couples and microstructural features that significantly change the corrosion rate. Devising strategies to mitigate this interfacial corrosion requires quantitative and correlative understanding of its surface electrochemical reaction. In this work, scanning electrochemical cell impedance microscopy (SECCIM) was employed to study location-specific corrosion in the interfacial region of dissimilar alloys, such as AZ31 (magnesium alloy) and DP590 (steel) welded using the Friction-stir Assisted Scribe Technique (FAST) processes. Herein, SECCM and SECCIM were used to perform correlative mapping of the local electrochemical impedance spectroscopic and potentiodynamic polarization to measure the effect of electronic and microstructural changes in the welded interfacial region on corrosion kinetics. Microstructural characterization including scanning electron microscopy and electron backscatter diffraction was performed to correlate changes in microstructural features and chemistry with the corresponding electronic properties that affect corrosion behavior. The variations in corrosion potential, corrosion current density, and electrochemical impedance spectroscopy behavior across the interface provide deeper insights on the interfacial region—which is chemically and microstructurally distinct from both bare AZ31 and DP590 that can help prevent corrosion in dissimilar metal structures.

## Introduction

High-resolution scanning probe techniques, such as scanning electrochemical microscopy (SECM) and scanning vibrating electrode technique (SVET), have been developed to study liquid-electrolyte interfaces and electron transfer kinetics. SECM uses a microelectrode immersed in an electrolyte to probe the electron transfer properties of a substrate, whereas SVET uses a vibrating microelectrode to measure potentiodynamic gradients above a surface. Since the microelectrodes in SECM and SVET are usually used at a constant height (especially for imaging), they are not as sensitive to changes in the faradaic current at grain boundaries or microstructure^1^. While some improvements to SECM increases resolution enough to reveal grain boundaries^2,3^, it has limitations due to diffusional broadening when imaging because the microelectrode does not contact the specimen^4^. Newly developed microscopic, droplet-based corrosion measurement techniques such as scanning electrochemical cell microscopy (SECCM) are used to capture localized or confined region electrochemical responses that can perform high resolution probing of grain boundaries, defects, and microstructures^5–8^. A microscopic probe (< 1 µm diameter) with a single- or double-barrel channel is filled with liquid electrolyte and used as tip for measurements. At the end of the tip, a droplet formed by surface tension is used as the substrate contact point. High-resolution probe techniques such as SECCM have advantages of recording electrochemical signals from microscopic features in metallic materials such as grain orientation, grain boundaries, second phases, and specific precipitate properties, which allow very location-specific measurement^5,9,10^. Furthermore, SECCM offers controlled substrate/electrolyte exposure time, which is especially important for samples prone to corrosion^11,12^.

Friction stir welding (FSW) is a solid-phase joining technique widely used to join similar and dissimilar materials^13–23^. At present, FSW is used in industries such as automobile, aerospace, shipbuilding, and railways^24–26^. Recently, efforts have been made to join dissimilar materials with a large melting temperature difference, such as Al alloys to steel^27^ and Mg alloys to steel using a novel friction-stir-assisted scribe technique (FAST)^23^. In this process, a scribe at the end of a tool tip develops mechanical features to join two materials. During the FAST process, a series of microstructural zones develop across the welded region, including the stir zone (SZ), thermomechanically affected zone, and heat affected zone. Each zone undergoes a different thermomechanical process and develops distinct grain structure, dislocation density, distribution of second phases, and precipitate characteristics. Furthermore, microstructural features from corresponding zones determine the individual corrosion susceptibilities^28,29^. Intense shear force and friction heating cause the SZ to develop the most refined and complex microstructure, which further complicates corrosion behavior in the SZ^30^. Several studies have been conducted to examine the corrosion properties of Mg and steel joints, which mainly used bulk Potentiodynamic (PD) polarization techniques to study the corrosion resistance of the entire sample containing interfacial region. Gupta et al*.* investigated the corrosion properties of gas tungsten arc welding (GTAW) alloyed to 304L austentic stainless steel. Austentite and ferrite phases were observed in the weld zone with varying volume fractions, which lead to different corrosion properties (i.e. corrosion resistance and pitting)^31^. Sidhu et al. used bulk corrosion techniques to understand the effect of the FSW process on the joint of Al and Mg alloys, which suggested that FSW techniques yields higher corrosion resistance based of the PD polarization curves^32^. Zhang et al. looked at the effects of Ni coatings on DP590 and 304 stainless steel and found an increase in corrosion resistance^33^. These studies suggest that the joining portion of the two similar metals have distinct electronic properties, that affect the joined metal as a whole. Kim et al. studied the effects of Al addition on friction stir welded AZ31B joints and found 55% improvement of joint corrosion resistance, where corrosion resistance was evaluated through H_2_ collection and bulk PD technique^34^. These authors suggested that the continuous formation of in-situ Mg_17_Al_12_ particles and their distribution along grain boundaries may have contributed to improved corrosion resistance. However, no electrochemical data of the grain boundaries with presence of Mg_17_Al_12_ particles was provided to support the postulation.

While microscale techniques such as SECM^35^ give more insights to localized corrosion behavior, the entire sample needs to be immersed in the electrolyte solution in which is it is hard to profile corrosion behavior as a function of time. Since the intrinsic microstructural character of each process zone determines its electrochemical response, SECCM-based local probing is appropriate for characterizing different corrosion/electrochemical responses from each process zone to identify the accurate corrosion mechanism and allow us to design new corrosion resistance materials for various structural applications^36–38^. For instance, Salleh et al.^28^ recently measured a higher corrosion rate of AM60 alloy in the SZ zone compared to AM30 alloy and were able to confirm process-zone–dependent corrosion behavior. In their work, the authors hypothesized that faster corrosion in AM60 could be influenced by galvanic coupling of cathodic β-Mg_17_Al_12_ within the matrix. The potential of Fe is known to be much higher than that of Mg, and therefore galvanic corrosion is inevitable in Mg-steel lap joints^29,39^. Galvanic corrosion is one of the major drawbacks to using Mg-steel mixed parts for automobile structural components^40,41^. Moreover, because microstructure and galvanic potential affect the corrosion behavior of dissimilar Mg-steel weld joints, their corrosion behavior is more complex than those of same material joints^42^. However, few studies exist on corrosion behavior of joints of Mg to steel, which is investigated herein. Recently, Scanning Electrochemical Cell Impedance Microscopy (SECCIM), a combination of SECCM, Electrochemical Impedance Spectroscopy and distribution of relaxation time (DRT) analysis were used to study interfacial events such as charge transfer and evolution of resistive oxide films on Mg surface^38^. In this work, we successfully used SECCM and SECCIM to study interfacial corrosion of dissimilar metal alloys joined by FAST processes. Galvanic differences between materials make preventing interface corrosion of dissimilar materials a challenge. To investigate interfacial corrosion behavior in multi-material systems, a local probe with a microliter-volume (electrolyte) droplet is used to collect electrochemical signals in a confined region. Although there are extensive studies available related to independent bulk corrosion behavior of Mg, Mg alloys and steels, interfacial corrosion behavior of multicomponent structural materials is difficult to predict using single-material data. Because the galvanic potential difference of Mg and steel is high, corrosion would be very severe in the Mg-steel joint region. Therefore, there is a strong need to understand the interfacial corrosion in these joints, so that pathways can be developed to prevent such corrosion to protect multi-material structural components. This study aims to demonstrate the SECCM/SECCIM-based probing technique to understand microscopic corrosion of a Friction-stir Assisted Scribe Technique (FAST) welded Mg-steel joint sample across the interface. These localized measurement along with the global electrochemical experiments may be used as input for multi-physics–based modeling tools to eventually predict corrosion behavior in these systems. The SECCM/SECCIM probing technique is also useful to measure the efficacy of mitigation strategies by revealing their effect at a local level.

## Experimental procedure

### Surface characterization of joint sample

AZ31 (3 mm thick) and DP590 (1 mm thick) were selected as dissimilar metal alloys to be joined using FAST. Welding parameters were 1950 rpm rotation speed and 0.75 m/min traverse speed and welded as lap shear configuration. After welding, interfacial microstructure and compositional analysis of the welded sample was characterized by scanning electron microscopy (SEM) and energy dispersive spectroscopy (EDS). Microstructural and elemental analyses were performed using a JEOL 7600 field emission SEM (FESEM) equipped with an Aztec Oxford Instruments Oxford Ultim Max 170 mm^2^ EDS detector and INCA Microanalysis software. A 20 kV accelerating voltage and 9.1 mm working distance were used for EDS analysis. Electron backscatter diffraction (EBSD) analysis was carried out using a JEOL 7600 FESEM with Oxford Symmetry EBSD Camera. EBSD mapping was performed at a working distance of 25 mm using an accelerating voltage of 20 kV and probe current setting of 16. Acquisition speed and step size were 18.4 Hz and 0.2 µm, respectively. Samples were 10 mm long, 3 mm wide and ~ 1 mm thick.

### SECCM/SECCIM setup and electrochemical measurements

SECCM/SECCIM and microscopy analyses were performed across the cross section of the joint material, from Mg side to steel side. Line and area scans were carried out on Mg-steel samples across the interface. A SECCM probe consisting of a pulled theta-glass capillary with ~ 30 µm tip diameter was used for the measurements during both line and area scans. A Sutter P-2000 laser puller was used to pull the 1 mm outer diameter, borosilicate theta capillary to ~ 30 µm. A narrow tip (1–5 µm) was initially used but yielded unsuccessful scans when significant change in humidity caused precipitation at the tip. Both sides of the theta capillary were filled with electrolyte containing ~ 10 µL of 0.01 M NaCl solution (pH = 7) using a microinjector at the room temperature (25 °C). For the SECCM corrosion studies performed in this work, 0.01 M NaCl electrolyte was chosen due to its lack of precipitation at the tip (which blocks conductivity) and stable formation of a menisucus at the end of tip as compared to 0.6 M (3.5wt%) which formed salt precipitate at the tip within an hour of measurement has started. Therefore, the dilute concentration 0.01 M NaCl was used which helped with avoiding the precipitation at the tip for the prolonged period of measurement which is particularly need for continuous XY area scans. Additionally, the lower concentration of 0.01 M may have lower corrosion rate compared to the measurement with 3.5 wt% NaCl over time, we expect that relative change in the intrinsic corrosion properties across the joint region, such as open circuit potential (OCP) and corrosion potential (E_corr_) determined by Tafel polarization should be similar for different NaCl the concentration. Since the focus of this work is study the relative changes in the intrinsic corrosion properties across the joint region of Mg-steel joint sample, the trends in OCP and corrosion potential mapping obtained in this work could be compared to conventional testing condition using 3.5 wt% NaCl. Sufficient care was taken that no air bubbles blocked either channel of the capillary probe. This was further ensured by forcing air through the filled capillary pipette using a syringe. The electrolyte level was also kept equal in both channels. Roughened Ag wire of diameter 0.25 mm was inserted into one barrel of the capillary tube to serve as a reference electrode (RE), and Pt wire of diameter 0.15 mm was used in the other side as counter electrode (CE) in the SECCM setup. In order to ensure the circuit was completed in such that the liquid at the end of the capillary tube made sufficient contact to the substrate, the current between the tip and the substrate surface was continuously monitored while the tip is approaching the surface. Once the tip made contact, a sharp spike in current is observed and the tip movement is stopped automatically, which indicated the liquid meniscus made contact to the substrate. The ends of the inserted wires were also kept at the same level. Optical microscopy was also performed after the SECCM/SECCIM measurement to locate the measurement footprints that correlate with electrochemical data obtained. The FAST welded Mg-steel joint sample acted as a working electrode (WE). All SECCM measurement was performed at room temperature (25 °C) with the relative humidity of 60%.

In a typical SECCIM measurement, the capillary probe filled with electrolyte and the RE/CE is moved down toward the sample and the tip movement stops as soon as the meniscus at the end of capillary probe contacts the WE and thereby completes the electrochemical cell circuit for performing measurements. A customized SECCM platform was purchased from HEKA (PG618 USB double amplifier) and used to perform localized corrosion measurements presented in this work. It consists of an XYZ positioner and controller, a potentiostat/galvanostat, a waveform generator, and a double current amplifier. The sample was mounted on the XY positioner.

At each point where the SECCM/SECCIM probe landed on the WE, electrochemical corrosion was studied by measuring potentiodynamic polarization and by electrochemical impedance spectroscopy (EIS). All electrochemistry testing was done within the confined contact area of a meniscus on the sample surface and no electrolyte leaks were observed. OCP was measured for 3 min before potentiodynamic polarization and EIS measurement, which our previous work has shown to be enough time for the system to stabilize^43^. For the Tafel curve, measurement direction was cathodic to anodic, with sweep rate 20 mV/s. A faster scan rate has been chosen since equilibrium is achieved sooner due to the lower electrolyte contact area, which is consistent with previous studies^7^. To observe the potential distribution and corrosion behavior, these measurements (OCP, Tafel and EIS) were performed at eight points across the interfacial area of the AZ31-DP590 joint. Subsequently, an area scan of a 70 µm × 500 µm region across the interface with step size of 30 µm was performed. During this area scan, OCP, Tafel, and EIS measurements were performed at individual points across the area, and data collected from individual points were used to construct the maps. For a rapid acquisition of impedance that resembles different resistive elements, data were recorded for only four frequencies—10 kHz, 1 kHz, 10 Hz, and 1 Hz—and used to determine the changes in impedance in each point. For the point analysis, the EIS measurement was performed between the frequency range of 1–25,000 Hz with 10 points per decade. An sinusoidal amplitude of ± 10 mV was maintained for all EIS measurement (both point and area scan) presented in this work, which is consistent with previously reported work^44^.

## Results and discussion

### Interfacial microstructural characterization

**Figure 1:**
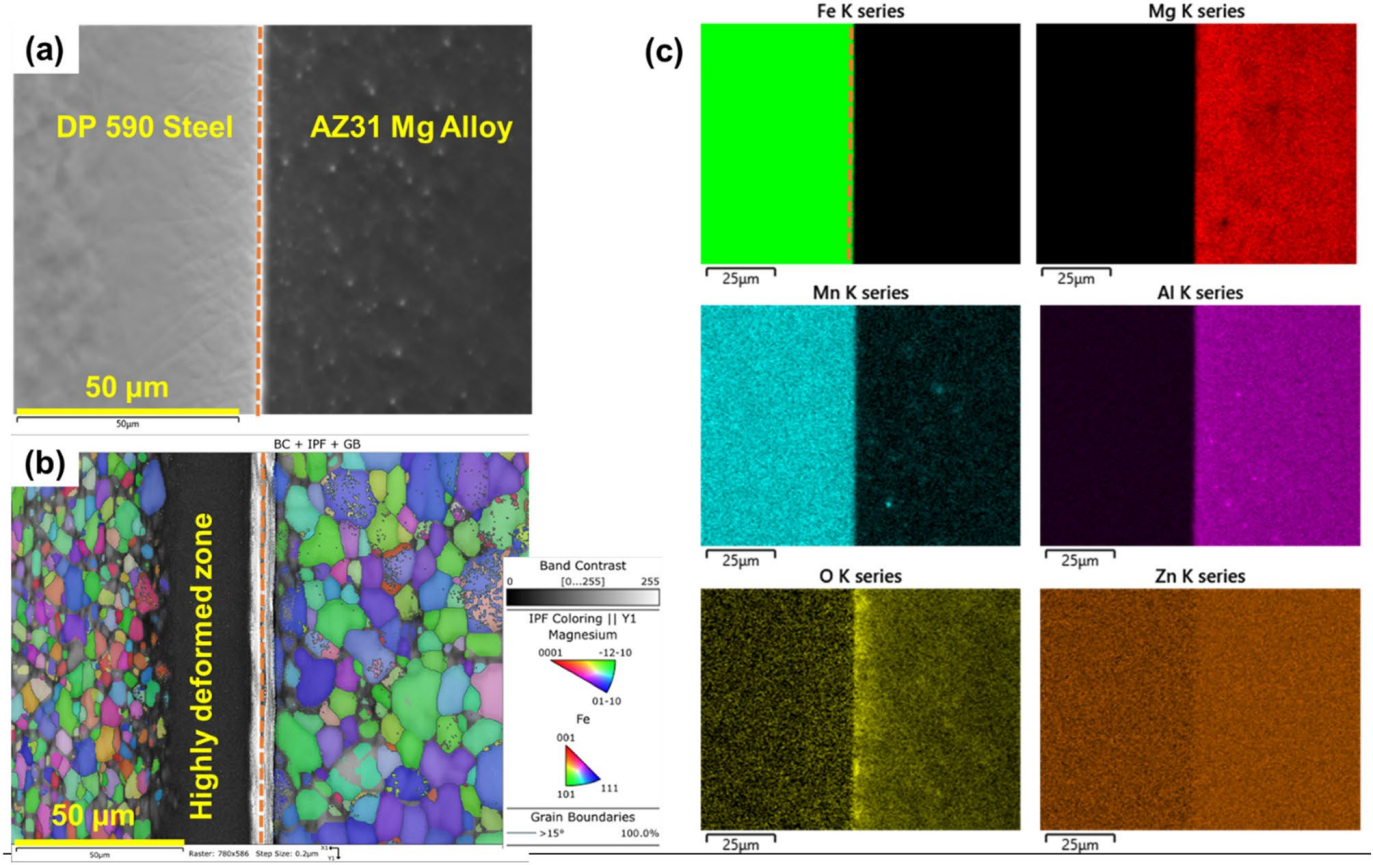
(**a**) SEM microstructure of FSW welded AZ31-DP590 steel. (**b**) EBSD map in interfacial region and (**c**) EDS maps of region used in area scan measurement.

Figure 1a shows the SEM microstructure of the welded Mg-steel FAST region. Microstructure shows a clear interfacial region between the steel and Mg alloy. On the Mg side, microstructure from EBSD shows recrystallized and equiaxed grains 10 ± 3.6 µm across (Fig. 1b). However, the steel side shows initially deformed microstructure near the interfacial region for approximately ~ 20 µm and fully recrystallized microstructure farther away with 4.4 ± 1.9 µm grain size. The steel side near the interfacial region was not indexed because the microstructure was highly deformed. To study the post-welding elemental distribution, EDS analysis was performed at multiple locations across the interface. Figure 1c shows the EDS map in the interfacial region. The EDS data show the Fe, Mg, Al, Zn, and Mn distribution and O enrichment in the interfacial region. The oxygen map indicates presence of oxide phases on the Mg side. Recently, Das et al.^45^ reported presence of oxide phase on the Mg side of a Mg-steel interface in a FAST welded sample.

### SECCIM point mapping of Mg-steel interface

**Figure 2:**
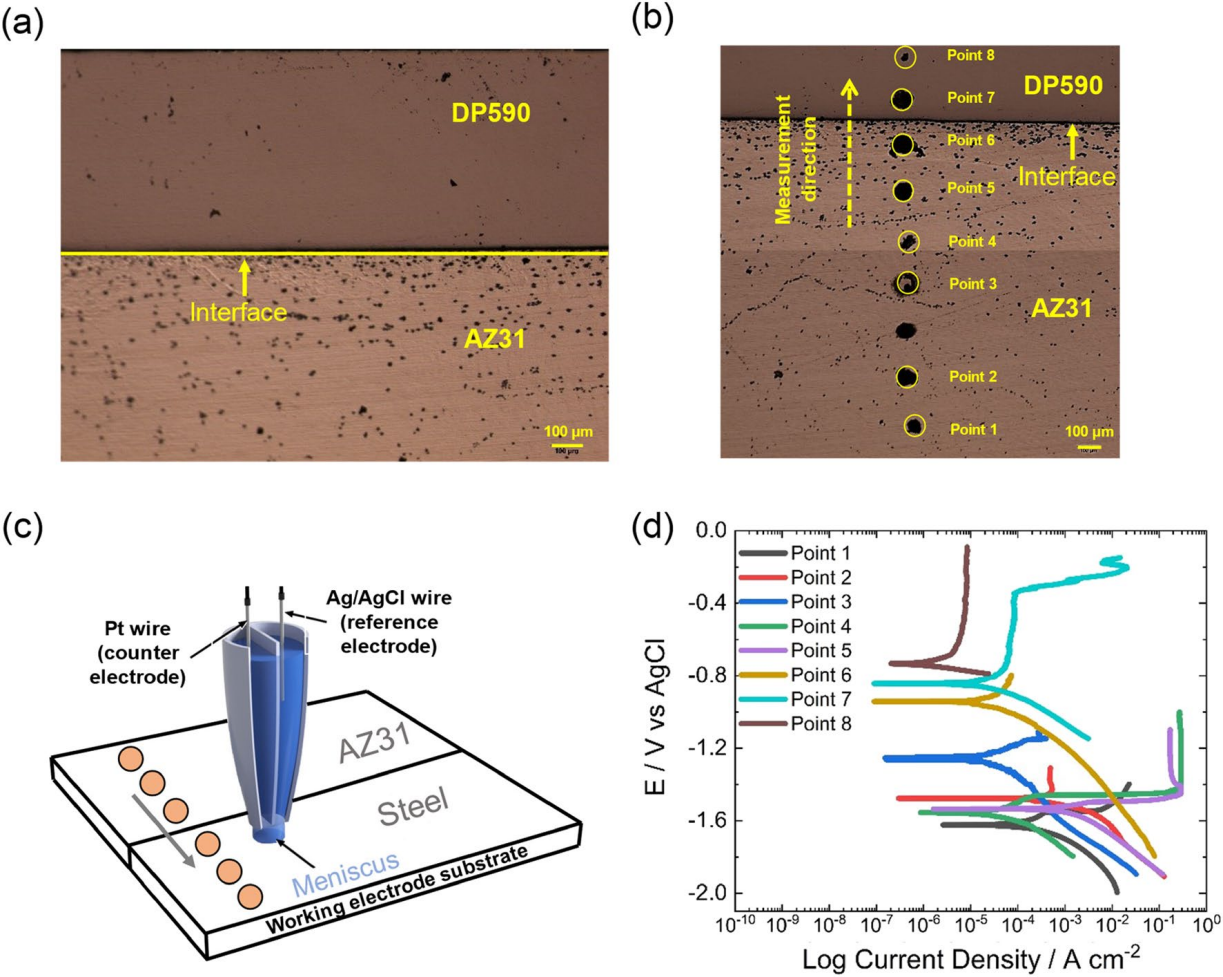
(**a**) Cross-sectional image of Mg-steel joint sample before SECCM/SECCIM experiment (dotted line indicates interface) and (**b**) image after SECCIM analysis; yellow circled areas are measurement “footprints” to indicate where the SECCM/SECCIM measurements were performed. (**c**) Schematic of SECCM/SECCIM setup used in this study. (**d**) Tafel plots for the corresponding points.

The characterization of the AZ31-DP590 steel welded region clearly revealed the presence of distinguishable Mg-oxide phases around the interfacial region; these are expected to play an important role in influencing the galvanic potential at microscopic length scales. To study in depth the galvanic potential drop in the interfacial region, we carried out SECCM/SECCIM measurements, which provided location-specific electrochemical activity information. Initially, the scan was performed at eight points across the interface. Figure 2a,b shows the optical-scale microstructure of a welded sample before and after SECCM/SECCIM measurements. The interfacial region is clearly visible, and distinguished using the yellow dotted line in Fig. 2a. After SECCM/SECCIM, measurement footprints were identified and marked using yellow circles, as shown in Fig. 2b. SECCM/SECCIM was carried out from the Mg to the steel side across the interface. Each yellow circle is ~ 30 µm wide. Points 1 to 6 were on the Mg side, and Points 7 and 8 were on the steel side. As per the micrograph, Points 1 and 8 are considered far from the interface and represent individual properties of AZ31 and steel, respectively, while Points 6 and 7 are close to the interface, and represent near-interface electrochemical properties of AZ31 and steel, respectively. Figure 2c is a schematic of the theta capillary probe including electrodes and electrolyte with a meniscus landing on the Mg-steel joint sample; it also indicates the measurement direction used to obtain the potentiodynamic polarization curves shown in Fig. 2d.

To obtain quantitative parameters of the galvanic potential differences across the interface, the corrosion potential (*E*_corr_), corresponding current density (*i*_corr_), and Tafel slope values were obtained from the Tafel plots shown in Fig. 2d and are listed in Table 1^46^. The obtained *E*_corr_ values from SECCIM for Mg (Points 1–4) and steel (Point 8) are similar to those reported for Mg/Mg alloys and for steels, respectively, in bulk cell experiments^47–51^. Point 3 shows a sudden drop in both *E*_corr_ (more noble) and *i*_corr_, which may be caused by impurities or sample artifacts. However, a clear transition of *E*_corr_ values was observed across the interface from the steel side to the Mg side, with E_corr_ values increasing in nobility. In fact, *E*_corr_ increased from − 1.53 V to − 0.94 V (vs. AgCl) from Point 5 to Point 6, which is near the interfacial region and indicates electronic properties are changing on the substrate. The *i*_corr_ value decreased by two orders of magnitude, which indicates the steel side is more corrosion resistant than the Mg side and signifies formation of a well-defined local galvanic couple. Furthermore, near the interface, the Mg side becomes more cathodic than the base material. However, the steel side shows the opposite phenomenon: the *E*_corr_ value near the interface (Point 7) becomes more anodic than Point 8 (base material). The Tafel slopes shown in Table 1 also indicate formation of a galvanically graded interface from the magnesium to the steel side. The Tafel slope increases from Points 1–3 to Points 4–5, which may indicate accelerated corrosion in the deformed zone close to the interfacial region. The steel side, however, has lower Tafel slopes than the Mg side, which is expected, since steel is more conductive and more corrosion resistant. The potential differences near the interface between Mg and steel dropped significantly (Table 1) which is in line with the corresponding change in Tafel slopes. The decrease of potential difference and increase in Tafel slope values near the interface compared to those for base material is a good indication of improvement in corrosion resistance, as observed by reducing galvanic potential differences between two dissimilar materials. Similarly, the *i*_*corr*_ of the Mg side also dropped significantly near the interface, which is also a good indication of improved corrosion properties (Table 1).

**Table 1:** Corrosion potential (*E*_corr_), corrosion current density (*i*_corr_), and corresponding Tafel slopes in the anodic and cathodic regions.

Locations	*E*_corr_ (vs. AgCl), V	*i*_corr_ (A/cm^2^) × 10^−7^	Tafel slope (anodic) mV/dec*	Tafel slope (cathodic) mV/dec
Point 1	− 1.62	2.49 × 10^−5^	9.2	− 13.3
Point 2	− 1.48	4.52 × 10^−5^	6.1	− 14.4
Point 3	− 1.26	8.06 × 10^−5^	7.8	− 13.1
Point 4	− 1.56	1.48 × 10^−5^	14.5	− 13.3
Point 5	− 1.53	1.66 × 10^−4^	24.1	− 13.01
Point 6	− 0.94	7.78 × 10^−6^	5.7	− 11.0
Point 7	− 0.84	4.97 × 10^−5^	5.6	− 13.2
Point 8	− 0.73	9.61 × 10^−7^	4.5	− 26.5

*dec decade.

### SECCM area mapping of Mg-DP590 interface

**Figure 3:**
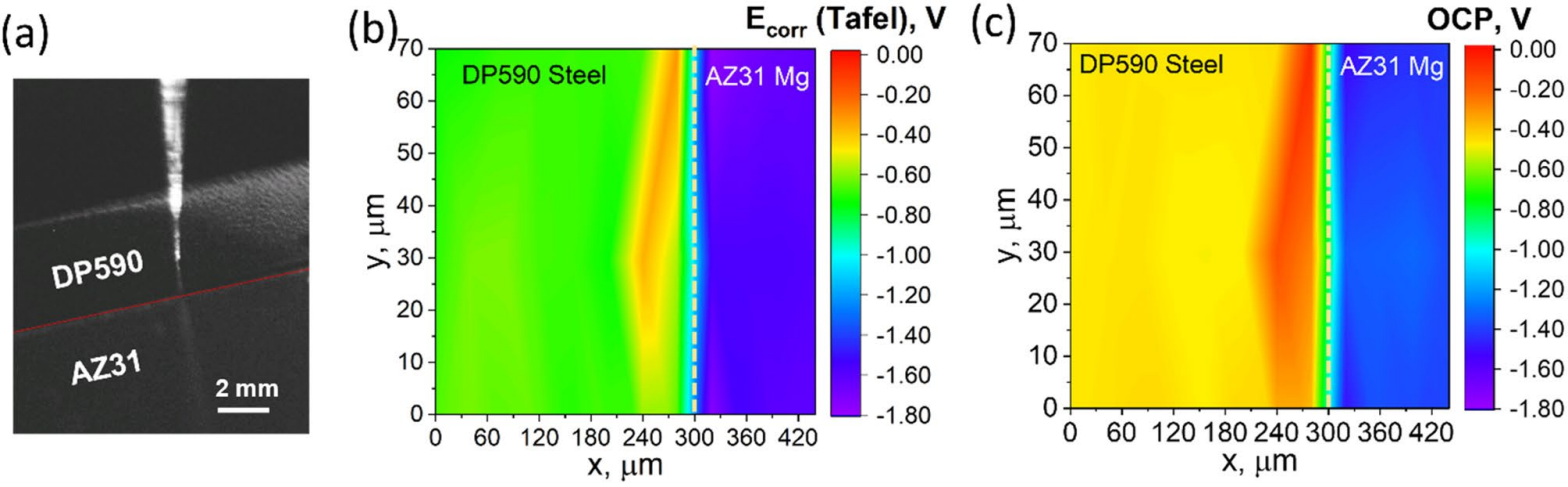
(**a**) Optical microscope image showing the positioning of the SECCIM electrode over the DP590 substrate, interface, and AZ31 regions as marked; (**b**)* E*_corr_ and (**c**) OCP *x*–*y* images of a 70 µm × 500 µm area are shown for the interfacial region. Dotted lines represent the interface between DP590 and AZ31.

**Figure 4:**
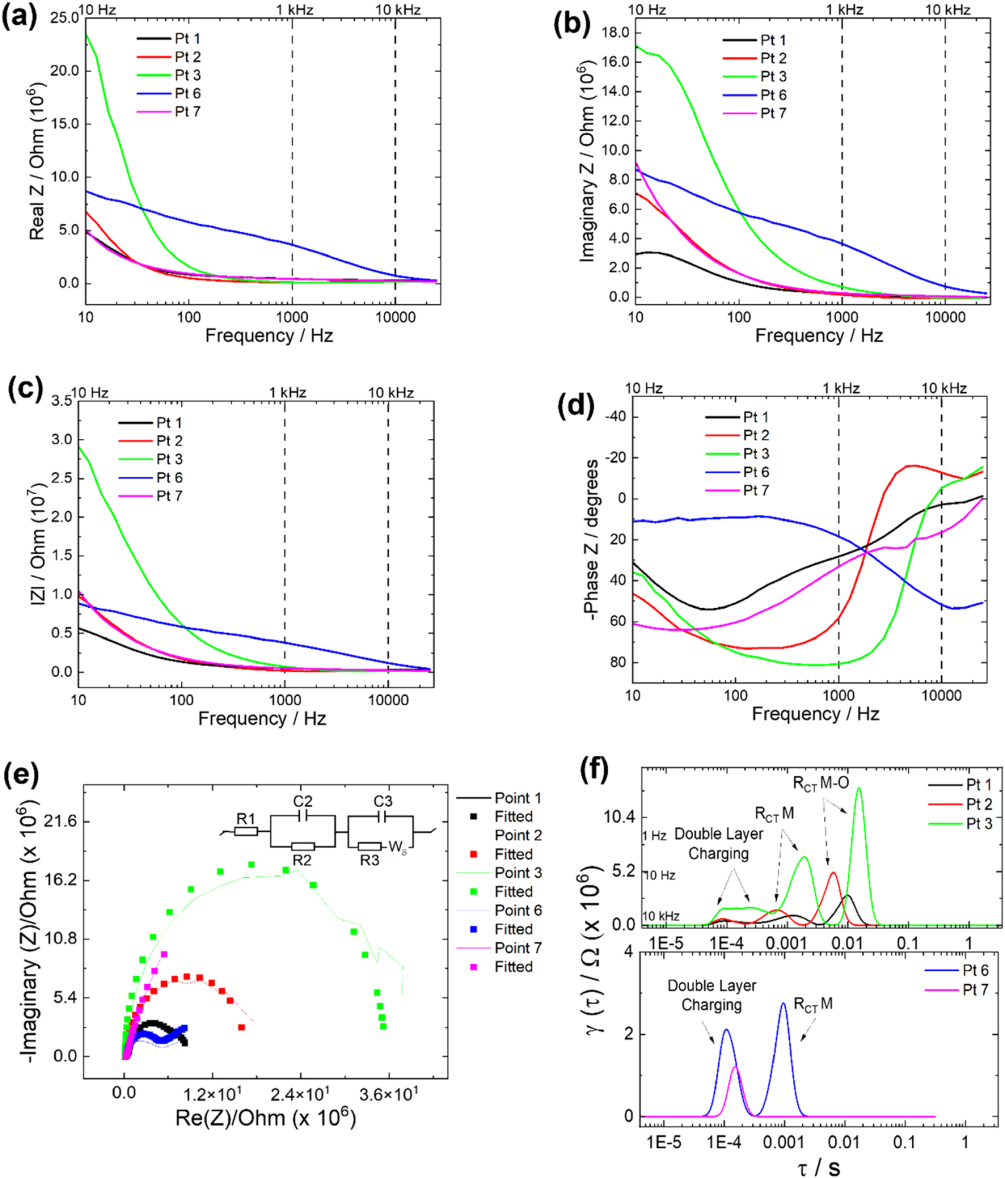
AC impedance (*Z*) measurements for Points 1, 2, 3, 6, and 7. Bode plots representing (**a**) real *Z* vs. frequency, (**b**) imaginary *Z* vs. frequency, (**c**) absolute *Z* vs. frequency, and (**d**) phase *Z* vs. frequency are shown to corroborate the Nyquist plots shown in (**e**) with the equivalent circuit. The frequencies used for EIS imaging are shown with dotted lines. Panel (**f**) shows the DRT results that can be correlated to specific electron transfer surface events in the Nyquist plots in (**e**).

**Figure 5:**
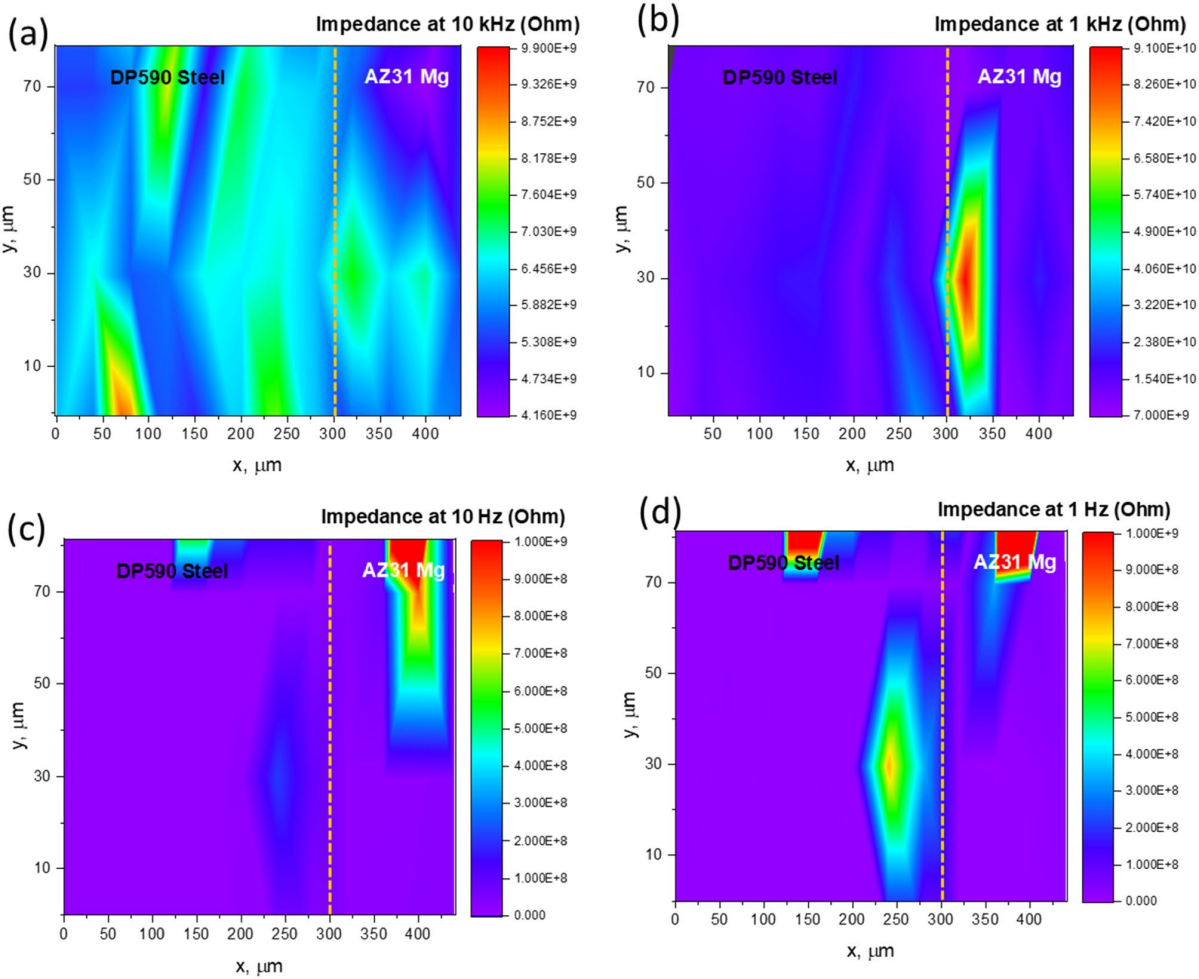
Area scan of EIS at four selected frequencies (10 kHz, 1 kHz, 10 Hz, and 1 Hz) measured along with the OCP and Tafel scans presented in Fig. 3. Dotted lines represent the interface between DP590 and AZ31.

After the location-specific SECCIM measurements, galvanic potential was mapped continuously across the interface to investigate changes in the interfacial region (Fig. 3). Figure 3b shows the *E*_corr_ values as a function of *x*–*y* position over a 70 µm × 500 µm region. The *E*_*corr*_ values became more noble from DP590 toward the interfacial region, which could indicate a thinner oxide layer (decrease in corrosion resistance) toward the welded region in the deformed zone. The OCP image (Fig. 3c) also indicates more noble values close to the interfacial region on the DP590 side, which supports the *E*_corr_ image and suggests a thinner oxide layer. The AZ31 Mg side has less noble *E*_corr_ and OCP values, which are likely due to increase in oxide layer thickness at the surface (also visible in the EDS data in Fig. 1c). Furthermore, a distinct interfacial region between the DP590 and AZ31 sides can be seen in both the *E*_corr_ and OCP images, likely resulting from the transition to the AZ31 side. The transition can also be observed in distinct changes in the impedance data (Figs. 4 and 5). The highly deformed region observed in the EBSD data in Fig. 1b can also be seen in the SECCIM results (Fig. 3b,c), where the OCP drops to 0 V vs. AgCl and *E*_corr_ increases to ~  − 0.4 V vs. AgCl.

### Point analysis of local electrochemical impedance response of the interface using SECCIM

To further explore the effects of an oxide layer formation near the interfacial region on relevant corrosion properties (e.g. evolution of protective layer), EIS was performed at the same locations as the point Tafel measurements shown in Figs. 2b and 3b. EIS is used to measure the impedance in response to applied sinusoidally alternating current through the WE and obtain information on diffusion and resistive elements present at or near the liquid-electrode interface. Distribution of relaxation times (DRT), an emerging EIS analysis method is also performed which allow us to examine a specific charge transfer events to be correlated to the Nyquist plots (shown in Fig. 4e)^52^. Figure 4a–d shows Bode impedance measurements and Fig. 4e shows a Nyquist plot for Points 1–3 (Mg side) and Points 6 and 7 (interfacial region). Results for Points 4–5 and Point 8 are shown in Figs. S1 and S2, respectively. Figure 4a and b show the real and imaginary parts of impedance as a function of frequency, respectively.

The Bode plot represents two distinct regions: (i) a DC plateau at high frequencies and (ii) a frequency-dependent region at lower frequencies, which arises from varying capacitance and resistive behavior of the joint interface^53^. The DC plateau in Points 1, 2, and 3 (on the AZ31 side) and Points 7 and 8 (on the DP590 side) indicate that capacitive behavior is consistent on both sides. The observed change in the capacitance is likely due to slight droplet size variation^44^ as well as changes in oxide distribution on the surface. The points measured in the highly deformed zone (Points 4 and 5) do not exhibit a DC plateau, which may be attributable to distinct surface chemistry observed in this deformed region. Point 6 (in the interfacial region) also does not have a DC plateau, which indicates that capacitive behavior in the interfacial region is different from those in either the AZ31 or DP590 side of the joint interface (also visible in the Nyquist plots in Fig. 4e, in a distinct diffusion region). Furthermore, the magnitude of the impedance (|*Z*|) shown in Fig. 4c shows trends similar to the real and imaginary parts of impedance. The phase angle as a function of frequency is shown in Fig. 4d, and represents the phase difference between the voltage and current for the measured spot. Points 1, 2, and 3 show similar profiles but differing minimum values at different frequencies, which is likely caused by changes in the chemical properties close to the deformed zone, which would in turn change the phase alignment (represented for Points 4 and 5 in Fig. S1).

The Nyquist plot of points 1, 2, and 3, corresponding to the AZ31 side, show resistance behavior that may correlate to an oxide layer (Fig. 4e). Impedance response of Points 4 and 5 (in the highly deformed region according to the EBSD results in Fig. 1b) is significantly different from those of Points 1–3. The inductance-like behavior at Points 4 and 5 (in the deformed zone) shown in Figs. S1 and S2 may indicate some metal ion dissolution during the measurement in this region, which could be a result of dissimilar metals at the joint^54^. Point 6 (also at the interface) does not exhibit the same inductance behavior as Points 4 and 5, which may indicate the chemical properties are changing close to the DP590 side. Point 8 exhibits less resistive behavior, which may indicate that an oxide layer is smaller in this region, causing an increase in the conductivity. The fitting parameters for curves in Fig. 4 are shown in Tables S1–S3.

Furthermore, DRT plots showed three events in Fig. 4e for Points 1, 2, and 3, which correspond to a minor double-layer charging region at the lowest time scale followed by a minor component that represents the metal charge-transfer resistance (R_CT_ M) and then a larger resistive component that shows oxide resistance (R_CT_ M–O), which were assigned based of the relative time scales observed in the DRT results. The DRP plot of Point 6 (at the interface) lacks an event that would correspond to *R*_CT_ M–O, which indicates that electronic properties are changing close to the substrate. Similarly, the DRT plot of Point 7 has only one event, which correlates to the time scale consistent with double-layer charging (also seen in a small resistance component in the Nyquist plot in Fig. 4e).

### Area scan analysis of local electrochemical impedance response of the interface using SECCIM

Figure 5 shows the area mapping of resistance values measured at four different frequencies (10 kHz, 1 kHz, 10 Hz, and 1 Hz) performed after Tafel and OCP measurement at each point. These resistance values may represent the resistive components of the electrode, surface oxides, double-layer resistance, and Warburg diffusion, respectively. Figure 5a shows the resistance mapping at 10 kHz without any distinct changes across the interface, which may indicate the presence of continuous electronic conductivity. Figure 5b shows distinctly lower resistance on the DP590 side and slightly higher resistance on the AZ31 side of the interfacial region, which corroborates the previous observation that the formation of an oxide layer produced by increased corrosion is more likely on the AZ31 side than the DP590. Furthermore, the presence of a lower resistance region in Fig. 5c, which corresponds to a double-layer capacitive element which may indicate the existence of a dissolution region caused by accelerated corrosion. The resistive component in Fig. 5d may be the attributed to a Warburg diffusion element, indicates lower diffusion resistance on the DP590 side of the interfacial region during measurement resulting from little or no formation of an oxide layer in that region. Overall, the area mapping of selected-frequency EIS showed changes in surface resistance likely due to corrosion happened after Tafel polarization. However, the resistance changes across the interfacial region is not well-defined which may be attributed to limited spatial resolution (~ 30 µm) of these measurements. However, the presence of increased resistance region near the interface may contributed from the more corrosion activity.

## Conclusions

Location-specific electrochemical corrosion properties were measured successfully using SECCM/SECCIM in a Mg-steel joint region. SECCIM results showed a significant drop in corrosion potential and current on the Mg side near the interfacial region. Impedance response also varied significantly in the joint region along with a higher Tafel slope; these suggest presence of heterogeneous chemical composition, as confirmed further by the EDS measurement. Overall, the difference in corrosion potential has dropped significantly in the interfacial region between Mg and steel, which is very beneficial for preventing galvanic corrosion resulting from dissimilar metals. The complex impedance analysis suggests variation the near-interface region and in the deformed region (according to the EBSD results), which is likely due to different chemical properties in these regions as compared to either the DP590 or AZ31 side. Furthermore, a significant drop in corrosion current was also observed on both sides near the interface. Our electrochemical measurements (Tafel and EIS) indicated that presence of a surface oxide layer on the Mg side could have contributed to such drops of corrosion potential and current in the interfacial region. In summary, SECCM/SECCIM has been demonstrated to be a useful location-specific technique to examine the interface between two dissimilar metals.

### Supplementary Information

Supplementary Information.

## Data Availability

The data that support the findings of this study are available from the corresponding author upon request.
