# Supplementary material for: Investigating electrochemical corrosion at Mg alloy-steel joint interface using scanning electrochemical cell impedance microscopy (SECCIM)
Source: Sci Rep. 2023 Aug 15;13:13250. doi: 10.1038/s41598-023-39961-2 (PMC10427676; doi:10.1038/s41598-023-39961-2)
Supplement: Supplementary file 1 — Supplementary Information. [file 41598_2023_39961_MOESM1_ESM.docx]

**Electronic Supplementary Information**

**Investigating Electrochemical Corrosion at Mg alloy-Steel Joint Interface Using Scanning Electrochemical Cell Impedance Microscopy (SECCIM)**

Venkateshkumar Prabhakaran^a,^*^,#^, Lyndi Strange^b,^*, Rajib Kalsar^b^, Olga A. Marina^b^, Piyush Upadhyay^b^, and Vineet V. Joshi^b^

^a^Physical and Computational Sciences Directorate, Pacific Northwest National Laboratory, Richland, WA, United States

^b^Energy and Environment Directorate, Pacific Northwest National Laboratory, Richland, WA, United States

*These authors contributed equally

^#^Corresponding author: [venky@pnnl.gov](mailto:venky@pnnl.gov), [vineet.joshi@pnnl.gov](mailto:vineet.joshi@pnnl.gov)

**Keywords:** Scanning electrochemical cell microscopy (SECCM); Dissimilar material; Friction stir weld; Mg-Steel Joint Interface; Localized Galvanic corrosion


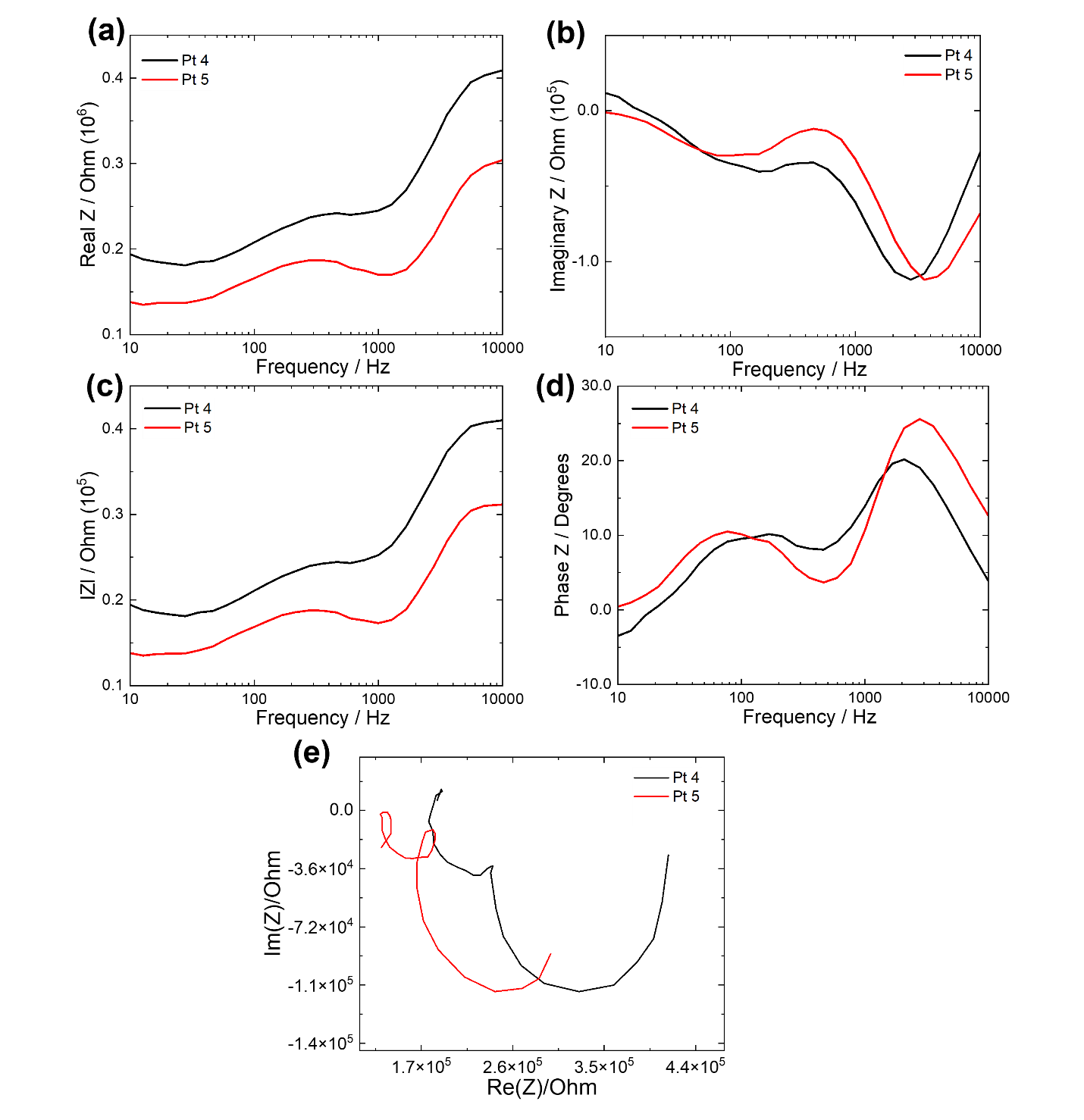


**Figure S1:** AC impedance measurements for Points 4 and 5, which correspond to the highly deformed zone in the electron backscatter diffraction image shown in Figure 1. Bode plots representing (a) real *Z* vs. frequency, (b) imaginary *Z* vs. frequency, (c) absolute *Z* vs. frequency, and (d) phase of *Z* vs. frequency are shown to corroborate the Nyquist plots shown in (e). Points 4 and 5 have different electrochemical impedance spectroscopy profiles from the other points taken in the line scan, which is expected since this region is deformed. Because the deformed region is complex, Nyquist plots were not created for Points 4 and 5.


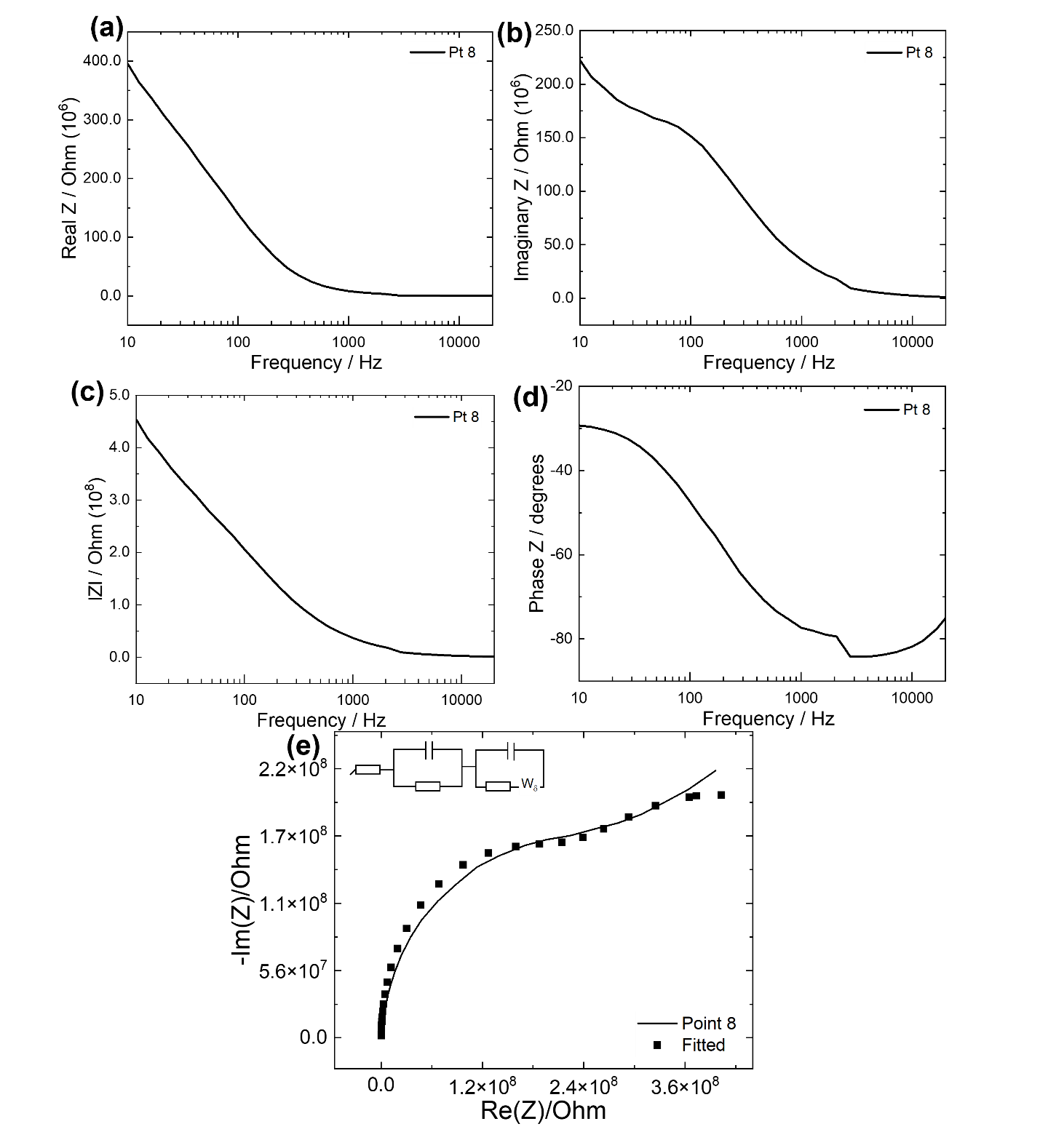


**Figure S2:** AC impedance measurements for Point 8. Bode plots representing (a) real *Z* vs. frequency, (b) imaginary *Z* vs. frequency, (c) absolute *Z* vs. frequency, and (d) phase of *Z* vs. frequency are shown to corroborate the Nyquist plot shown in (e). Point 8 is the last point measured in the line scan on the DP590 side and has a higher resistance than the other points on the line scan, which could result from a thicker oxide layer in this region.

**Table S1. Equivalent circuit fitting parameters for Points 1, 2, 3, 7, and 8**

|  | **R1 (ohm)** | **C2 (F)** | **R2 (ohm)** | **C3 (F)** | **R3 (ohm)** | **W_δ_  (ohm)** | **W_δ_’ (s)** |
| --- | --- | --- | --- | --- | --- | --- | --- |
| **Point 1** | 314.9 | 0.69 × 10^−9^ | 0.32 × 10^6^ | 1.5 × 10^−9^ | 5.7 × 10^6^ | 2.3 × 10^6^ | 0.17 |
| **Point 2** | 142.3 | 2.0 × 10^−9^ | 1.26 × 10^5^ | 1.97 × 10^−9^ | 3.8 × 10^6^ | 17.34 | 6.2 × 10^8^ |
| **Point 3** | 131.0 | 2.8 × 10^−10^ | 3.53 × 10^5^ | 30264 | 5.562 | 1.2 | 0.02 |
| **Point 7** | 213.1 | 1.5 × 10^−9^ | 26.3 × 10^6^ | 1.7 × 10^−10^ | 139.8 | 1.4 × 10^6^ | 6.45 × 10^9^ |
| **Point 8** | 287.3 | 6.5 × 10^−12^ | 0.23 × 10^9^ | 4.16 × 10^−8^ | 3.0× 10^5^ | 4.8 × 10^8^ | 1.3 |

**Table S2. Equivalent circuit fitting parameters for Point 6**

|  | **R1 (ohm)** | **Q1  (F s^(a-1)^)** | **R2** | **Q2  (F s^(a-1)^)** | **a** | **R3 (ohm)** | **W_δ_ (ohm)** | **W_δ_” (cm^2^/s)** |
| --- | --- | --- | --- | --- | --- | --- | --- | --- |
| **Point 6** | 38.6 | 3.74 × 10^−8^ | 2.83 × 10^7^ | 3.76 × 10^−11^ | 0.95 | 3.5 × 10^6^ | 3.3 × 10^6^ | 3.10 × 10^−5^ |

**Table S3. Table of *Χ^2^* values for the EIS equivalent circuit fittings.**

| **Location** | **Χ^2^ / \|Z\|** |
| --- | --- |
| Point 1 | 0.475 |
| Point 2 | 2.813 |
| Point 3 | 2.040 |
| Point 6 | 0.761 |
| Point 7 | 0.578 |
| Point 8 | 1.559 |
